# Adolescents and Distance Learning during the First Wave of the COVID-19 Pandemic in Italy: What Impact on Students’ Well-Being and Learning Processes and What Future Prospects?

**DOI:** 10.3390/ejihpe11030052

**Published:** 2021-07-09

**Authors:** Elena Commodari, Valentina Lucia La Rosa

**Affiliations:** Department of Educational Sciences, University of Catania, 95124 Catania, Italy; e.commodari@unict.it

**Keywords:** adolescents, COVID-19, distance learning, wellbeing

## Abstract

Background: This study aimed to analyze the experiences related to distance learning in a large sample of students in upper secondary school during the first wave of the COVID-19 pandemic in Italy and to explore the potentialities of distance learning for students’ well-being. Methods: Between 22 April and 1 May 2020, 1017 students completed an online survey about the characteristics of online didactic, the availability of devices for distance learning, and the psychological experiences related to e-learning. Results: All Italian schools have adopted distance learning, even if each teacher employs different approaches to e-learning. Students are aware of the importance of distance learning for the containment of the pandemic and of the need to continue with online teaching to avoid the resumption of the infections. However, distance learning is associated with a significant increase in student workload and a consequent psychological distress related to homework. Specifically, students are more distracted in studying, have difficulty organizing study and are concerned that their school career may be adversely affected by the lockdown. Furthermore, a significant percentage does not perceive adequate support from teachers. Conclusions: Future studies will have to explore the impact of distance learning even in the subsequent waves of contagion, taking into account the persistence of the stress from the pandemic.

## 1. Introduction

From March 2020, many countries in the world are in some form of quarantine to contain the COVID 19 pandemic. Quarantine has periodically been used for centuries to contain and control the spread of infectious diseases [1,2,3,4]. Most productive and economic activities have been disrupted, public transportation has been reduced, and shops, restaurants, bars, gyms, and other recreational places have been closed.

The necessity of social and physical distancing to contain the COVID-19 pandemic has made the use of new technologies even more essential than in the past [5]. Indeed, people have to stay physically separated but are strictly connected through social networks and mass media.

This huge spread of media communication due to the pandemic is especially evident in the educational field. One of the main measures of most governments during this pandemic was the closure of the educational institutions in order to contain the spread of the disease. According to the United Nations Educational, Scientific and Cultural Organization (UNESCO), school closures are impacting over 90% of the world’s student population [6]. In many countries, governments have activated measures to maintain continuity of learning. These measures provide different forms of distance learning, which are characterized by the teacher-learner separation by space and time, and the use of media and technologies to enable communication and exchange during the learning process despite separation [6].

In Italy, schools and universities were first closed on March 6, 2020, and during this last year there have been reopenings and closures based on the trend of the contagion in the various Italian regions. According to the ministerial indications, schools are delivering education remotely, through a mix of technologies, such as online platforms, television, and social networks, to guarantee continuity of curriculum-based study and learning for the students. However, there is not a standard model of home-based distance learning. Some schools have attempted to reproduce the structure of a classic face-to-face lesson via the web, using learning platforms; others have chosen web-based exchanges using social media or e-mail. Moreover, the public television service has implemented the programming of academic contents to aid disadvantaged students who cannot use online school [7,8,9].

Several variables influence the effectiveness of distance learning strategies. The availability of digital learning platforms, the presence in the household of digital devices and internet connectivity, the students’ ability to use these instruments, adequate spaces in the home, and other variables such as the capacities of the teachers to use technologies and methodologies for activating and facilitate home-based learning are central for the good outcome of school at home [10,11]. However, it is highly presumable that the success of each form of distance school also depends on the psychological experiences of the students, the emotional responses that accompany the new daily routines and approach to the study, and the presence of disorders that may make learning more difficult, such as specific learning disorders or attention deficit hyperactivity disorder (ADHD) [9,10,12,13].

The present study aimed to investigate experiences related to home-based distance learning in a large sample of students of the upper secondary school in Italy during the first wave of the COVID-19 pandemic. In particular, the overall goal of this study was to analyze their beliefs and opinions on several aspects of the experience of distance learning and to explore the future potential of distance learning for students’ well-being.

## 2. Materials and Methods

### 2.1. Participants

The survey was addressed to students of the upper secondary school in Italy. The study sample included 1017 students (males = 352; females = 665) who attend upper secondary school, aged between 13 and 20 years (mean: 16.57 ± 1.20). The participants lived in 14 of the 20 Italian regions; 597 of the respondents lived in a provincial seat, while 420 lived in towns that are not the provincial seat. Teachers and some students collaborated in the recruitment of the participants, sharing an online survey on the leading social networks and inviting students to respond to the questionnaire.

### 2.2. Procedures

This study refers to a larger study—on the psychological experience of the COVID-19 pandemic in a sample of Italian adolescents—whose results have already been published in a previous article by our research group [14]. In particular, this article focuses on the experience of distance learning and the expectations on school measures after the lockdown.

An online survey was diffused by volunteer teachers and students between 22 April and 1 May 2020, during the first wave of the COVID-19 pandemic in Italy.

The study complies with the Declaration of Helsinki and with the Italian guidelines on research with human subjects and protection of privacy. No sensitive data that could identify the participants was collected. Parents gave their consent to the participation in the study in the case of underage students. The study was approved by the Internal Ethics Review Board (IERB) of the Department of Educational Sciences, University of Catania (7 January 2021).

### 2.3. Survey Instrument

The web-based survey used for this study was developed according to the Checklist for Reporting Results of Internet E-Surveys (CHERRIES) [15]. It comprised 81 multiple-choice and short-answer questions and collected sociodemographic information, such as age, gender, area in which the respondents live, type of upper secondary school, academic grade, number of persons in the household, and other information. Moreover, it explored perceived health risk related to COVID-19, knowledge and information on measures to control the pandemic, beliefs and opinions on stage two of the quarantine, and psychological experiences related to quarantine, as described in our previously published article [14]. Regarding their opinions and beliefs, participants were asked to indicate whether they agreed or disagreed with a number of statements regarding lockdown and resumption of activities, with particular reference to school activities. Regarding feelings, emotions, and moods, participants were asked to complete a Likert-type scale that focused on the personal feelings about one’s cognitive, physiological, and behavioral state. The psychological experience scale measures two aspects: “negative feelings” (alpha Cronbach = 0.81) and “positive feelings” (alpha Cronbach = 0.78). A high score corresponds to a high perception of negative or positive feelings, respectively.

For the purpose of this article, we have only reported data on the availability and characteristics of online didactic, the availability of devices for participation in the online school, the psychological experiences related to e-learning, and the opinions on the measures that school should activate after the lockdown. 

### 2.4. Statistical Analysis

Data were analyzed using the Statistical Package for the Social Sciences (SPSS) version 25.0 (IBM Corporation, Armonk, NY). Quantitative data were expressed as frequencies and percentages in the case of categorical and ordinal variables and as the mean and standard deviation in the case of continuous variables. Comparisons between continuous variables were made by *t*-test, while Pearson chi-square tests were performed to explore the associations between categorical variables. A *p*-value < 0.05 was considered significant.

## 3. Results

### 3.1. Demographic Characteristics of the Participants

Table 1 summarizes the demographic characteristics of our sample. For a more detailed description of our study sample, please refer to the article already published by our research group [14].

### 3.2. Organization of at Distance Learning

Table 2 shows the distance learning modalities during the first wave of the COVID-19 pandemic in Italy. All the participants were referred to do home-based distance school. However, the organization of online learning was not the same for all schools. Some schools have created virtual classrooms and maintained the previous organization of the school day; other schools have created virtual classrooms but have changed the overall duration of the school day and the number of school days in a week. The remaining schools did not use virtual classrooms, and each teacher provided independent learning materials to the students, using social networks or emails. The responses of the students also showed that there were differences in the different class groups of the same school, and in some cases, each teacher of a class group used a different organizational approach to distance learning. In detail, 592 students (58.3% of the participants) reported that the duration of the school week was not changed, while the remaining participants reported a different duration of school week compared to the pre-pandemic period. Moreover, only 49.1% of the participants took lessons online every day. A total of 98.1% of the students used an online platform (such as Google Class or Zoom) for distance learning. A total of 48.8% of the students reported that all teachers of their class group used a common approach to e-learning. Of these, 48.8% reported that their teachers organized the virtual lessons as a face-to-face lesson. Teachers did roll call, explained, assigned schoolwork, and interviewed students. In these cases, the students were not online in every moment of the school day in order to avoid tired eyes due to monitor. Besides, 48.2% of the participants reported that the teachers of their class group employed different approaches to e-learning, using the online resources they preferred. Only 1.8% of the students reported, instead, that their teachers used email or social networks to send learning materials, such as slides. In these cases, the students worked in autonomy at home. Eight students did not respond to this question.

Unfortunately, not all the students had a tablet or a notebook for online lessons: in this regard, 7.6% of the participants in the survey reported that they use their mobile phone, and the 27.4% of the students who had a notebook or a tablet shared it with another person in the family, during the school day. Moreover, 17.5% of the students took their remote lessons in a room in which there were other persons.

### 3.3. Beliefs and Opinions of the Students on Hypothetic Behavioral Measures the Reopening of Schools

Students were asked to express their agreement about a series of measures that could be useful to activate with the reopening of schools after the lockdown.

Table 3 reports the participants’ opinions on the organization of teaching activities after the lockdown. The majority of the students (82.6%) believed that not all activities will be able to resume as in the period preceding the pandemic. Only 17.4% of the interviewed believed that no measure will be needed. A total of 68% of the students believed that the school should continue at distance learning, while 54.8% believe that all the activities should continue online except the exams. A total of 276 students (27.1%) believed that the only activities in presence should be the school meetings in small groups. There were no differences by age and gender in the responses on the school measures for the next years. There was a significant association between attended school and opinions related to the reopening. More specifically, the students attending classical and scientific high school were more likely to continue distance learning than students of technical institutes (*χ*^2^ = 18.786, *p* = 0.009). No significant correlations were obtained between living or not in a red zone and opinions on the school measures necessary to contain the epidemic (*p* > 0.05). Furthermore, a chi-square test for association was conducted between the organization of distance learning and beliefs and opinions of the students on behavioral measures regarding the reopening of schools. All expected cell frequencies were greater than five. There was a statistically significant association between holding classes every day and preference for resumption of face-to-face classes as in the pre-COVID period (*χ*^2^(1) = 6.53, *p* = 0.011). More specifically, students who do not attend classes every day are more likely after than before the pandemic to resume classes in attendance. There was also a significant association between possession of an appropriate device to follow the lessons (PC or tablet) and preference for continuing the lessons at a distance (*χ*^2^(1) = 6.02, *p* = 0.014). In particular, students who do not have adequate connection devices are less likely to agree to continue distance learning. Finally, a significant association was detected between having to share a device in order to follow lessons and the preference for continuing lessons by distance learning (*χ*^2^(1) = 6.28, *p* = 0.012). In this regard, students who are forced to share their PC or tablet with other family members are less likely to continue with distance learning.

### 3.4. Experiences Related to Home-Based Distance Learning

The experiences of home-based distance learning in our sample are varied and are reported in Table 4. First of all, the overwhelming majority of the sample (86%) declared that the homework load has increased significantly since the pre-pandemic period. Consequently, a large percentage of the adolescents of our sample said they had little time to devote to themselves because they were always busy with their homework (65.3%). More specifically, females claimed to have less time for themselves than males (*p* = 0.002). Furthermore, adolescents living in a “red zone” declared they had more homework than before compared to their peers not living in a “red zone” (*p* = 0.025). No significant differences were found in relation to the school attended and other sociodemographic variables.

This overload of activities had a negative impact on the teenagers interviewed with regard to their ability to concentrate on studying and concerns about their school career. Indeed, 77.1% of the participants were more distracted during the study, 51.3% did not manage to study following the usual rhythms and 64.6% were concerned about falling behind in learning. Females tended to become more distracted during the study (*p* < 0.0001), had more difficulty studying at their usual pace (*p* = 0.033) and were more worried about their school career (*p* = 0.035) than males. Furthermore, adolescents living in a “red zone” were more worried about falling behind in scholastic learning than their peers not living in a “red zone” (*p* < 0.001).

However, despite concentration difficulties in the study, most of the students said that they were not studying less than usual during this period (54.1%). Furthermore, the teachers seem to be a point of reference for the teenagers in our sample. Indeed, 57.5% believe that in this period, the teachers were more understanding than usual. Interestingly, males tended to perceive teachers as more understanding than did females (*p* = 0.015). There are no significant differences related to the school attended or to other socio-demographic variables.

## 4. Discussion

The aim of this study was to capture a range of experiences of high school students during the first wave of the COVID-19 pandemic in Italy to better understand their needs and concerns about distance learning.

The novel coronavirus (SARS-CoV-2) pandemic has totally revolutionized the school system and teaching, not only in Italy but all over the world [6]. Distance learning has become the norm and all schools have had to use media and technologies to continue their educational activities [7]. In Italy, an intense debate is currently underway on the future of the many students of schools of all levels in order to plan the scenarios for the later stages of the pandemic [16].

The results of our study confirm that all Italian schools have adopted distance learning, even if, in most cases, the organization of the teaching activities is left to the free choice of each teacher and there are no shared standards and guidelines. Consequently, some teachers organize virtual lessons as a face-to-face lesson, others use email or social networks to send learning materials, such as slides. More generally, each teacher employs different approaches to e-learning, using the online resources they prefer.

Furthermore, distance learning is not simple for all the students interviewed: in this regard, a not-insignificant percentage of our sample did not have a personal computer with which to take lessons online or had to share the PC with other family members and follow the lessons while in a room with other people. More specifically, these variables were found to be significantly associated with students’ opinions on resuming face-to-face teaching activities or continuing with distance learning. Students who do not have adequate devices to follow online lessons or who have to share their devices with other family members are more reluctant to pursue distance learning activities and more likely to resume face-to-face learning activities. This is certainly an important fact to think about. Indeed, several studies underlined that the closure of schools and the adoption of distance learning due to the COVID-19 pandemics have in many cases contributed to further exacerbating situations of educational inequality related to disparities in opportunities and conditions [17]. Examples are students who do not have a device and/or an internet connection [18,19], or who experience particularly fragile conditions such as disabilities and complex family situations. Therefore, taking into account the need to continue distance learning due to the new waves of contagion, it is essential to implement policies that allow all students equal access to the information technologies required for online learning [20].

The students interviewed are aware of the importance of distance learning for the containment of the pandemic. Indeed, the majority of our sample is convinced that it will be necessary to continue with online teaching also in the phases following the quarantine to avoid the resumption of the infections.

However, as already highlighted in previous studies on this topic [21,22], distance learning has resulted in a significant increase in student workload and a consequent psychological distress related to homework. The adolescents interviewed complained of an excessive load of homework during this period compared to the pre-pandemic one with a consequent reduction of the free time to devote to themselves; furthermore, they tend to become more distracted in studying, have difficulty organizing study at their usual pace and are concerned that their school career may be adversely affected by this lockdown period. In particular, our results show that females suffer more from increased homework than males, confirming that gender is an important variable with respect to the impact of the pandemic on the school performance and psychological well-being of adolescents [14].

In this scenario, the role of teachers is of primary importance in promoting adequate ways of learning and in reducing students’ stress. In this regard, although 57.5% of the students interviewed said that their professors are more understanding in this period, there is a significant percentage that does not perceive adequate support from their teachers. These findings are in line with those reported by other studies showing a perceived lack of support from teachers among adolescents [22]. More specifically, in our sample, females tend to perceive a lack of support from teachers more than males, confirming that females report greater psychological difficulties in dealing with the impact of lockdown and distance learning [14]. Therefore, it is important to also provide adequate support for teachers in order to help them manage the difficulties of their students and create a student-centered learning environment in contrast to a teacher-centered approach [23,24].

This study is one of the first conducted in Italy about opinions, beliefs and experiences of Italian adolescents about the distance learning during the quarantine for COVID-19 pandemic. Furthermore, the sample is very large (more than 1000 students), ensuring a good generalizability of results.

Nevertheless, some limitations of the study should also be considered. First of all, the internet-based questionnaire used for this study may not have guaranteed complete accuracy in answering questions. Furthermore, some Italian regions are not present in our sample even though the three main areas (northern, central and southern Italy) are adequately represented.

## 5. Conclusions

In conclusion, the COVID-19 pandemic has opened an important and urgent issue concerning the impact of distance education on the learning processes and psychological well-being of students, in particular children and adolescents. Undoubtedly, these are unprecedented circumstances that can promote stress and anxiety in both students and teachers. For this reason, the impact of these new modalities of distance learning is a very urgent issue to be addressed. In this scenario, the youth population must be adequately supported in coping with psychosocial consequences of school closures and distance learning.

Future studies will have to explore the impact of distance learning, even in the subsequent waves of contagion, in order to adapt the organization of teaching according to the needs of students and taking into account the persistence of the stressful event of the pandemic. Furthermore, it is important to investigate the impact of distance learning on the situation of educational inequalities, such as the lack of a device and/or an adequate internet connection, in order to prepare interventions that reduce as much as possible the educational disparities linked to the new modes of distance learning. Finally, it will also be interesting to assess the impact of distance learning on teachers and parents in order to identify the main difficulties and plan appropriate support interventions.

## Figures and Tables

**Table 1:** Sociodemographic characteristics of the participants.

		n	%
Gender	Female	665	65.4
	Male	352	34.6
Age (years old)	13	7	0.7
	14	58	5.7
	15	98	9.8
	16	309	30.4
	17	296	29.1
	18	211	20.7
	19	28	2.8
	20	10	1
School	High school	814	80.0
	Technical institute	153	15.1
	Professional institute	50	4.9
Area	Chief town	597	58.7
	Not chief town	420	41.3
Region	Lombardy	46	4.5
	Piedmont	12	1.1
	Trentino Alto Adige	2	0.2
	Friuli Venezia Giulia	2	0.2
	Emilia Romagna	23	2.2
	Abruzzo	5	0.5
	Molise	7	0.7
	Toscana	9	0.9
	Umbria	46	4.5
	Lazio	351	34.5
	Campania	83	8.2
	Puglia	17	1.7
	Sicily	414	40.7
“Red zone”	Yes	310	30.5
	No	707	69.5
Size of the household	1	8	0.8
	2	78	7.7
	3	258	25.4
	4	469	46.1
	>4	204	20.1
Affected by COVID-19	Yes	5	0.5
	No	876	86.1
	Uncertain	136	13.4
Family member with COVID-19	Yes	30	2.9
	No	987	97.1
Family member working with COVID-19 people	Yes	102	10.0
	No	915	90.0

**Table 2:** Distance learning modalities during the COVID-19 pandemic.

	n	%
The duration of the school week remained the same	592	58.3
Lessons are held regularly every day, with all the teachers, as before the COVID-19 pandemic	499	49.1
The school carries out distance learning using a specific online platform (e.g., Google Class or Zoom)	996	98.1
The student has a computer, a tablet or a notebook to use for online lessons	938	92.4
The student does not share the use of the tablet or the notebook with other people	700	72.6
The student follows online lessons in a room alone	837	82.5

**Table 3:** Study participants’ opinions on the organization of teaching activities after the lockdown.

	Agree		Disagree	
	n	%	n	%
Resume all teaching activities regularly as in the pre-Covid period	177	17.4	840	82.6
Continue with all distance learning activities, as is being done in this period of quarantine	692	68.0	325	32.0
Continue with distance learning activities except for exams	557	54.8	460	45.2
Continue with distance learning activities with the exception of the meetings of the collegial organs (e.g., class councils, assemblies, etc.)	276	27.1	741	72.9

**Table 4:** Differences in distance learning experiences in the study sample.

	Male (n = 352)		Female (n = 665)			Not living in a “Red Zone” (n = 707)		Living in a “Red Zone” (n = 310)		
In This Period of Distance Learning, the Student …	M	SD	M	SD	*t*-Test	M	SD	M	SD	*t*-Test
… has many more homework than before	3.77	1.25	3.93	1.13	1.90	3.82	1.18	4.00	1.14	2.24 *
… is always busy with the homework	2.93	1.27	3.20	1.29	3.15 **	3.06	1.28	3.22	1.31	1.78
… is studying less than usual	2.57	1.42	2.58	1.46	0.09	2.54	1.45	2.64	1.44	0.94
…is get distracted during study	3.28	1.27	3.60	1.25	3.77 **	3.47	1.27	3.53	1.27	0.66
… can study regularly following the usual rhythms	2.68	1.28	2.51	1.23	−2.13 *	2.60	1.27	2.49	1.17	−1.29
… is worried about being left behind with learning	3.07	1.46	3.28	1.49	2.12 *	3.08	1.47	3.48	1.47	3.95 **
… thinks that the professors are more understanding than usual	2.91	1.25	2.71	1.19	−2.44 *	2.79	1.20	2.76	1.25	−0.37
… has difficulty organizing the day	2.67	1.26	2.89	1.36	2.49 *	2.71	1.32	3.05	1.31	3.66 **
… checks if he/she uses the time effectively	2.86	1.23	2.76	1.31	−1.08	2.79	1.29	2.81	1.27	0.22
… organizes an adequate and orderly study environment	3.06	1.36	2.98	1.32	−0.82	3.07	1.32	2.88	1.37	−2.07 *

* *p* < 0.05; ** *p <* 0.01. Note: M = Mean; SD = Standard Deviation.
